# Human 8-oxoguanine DNA glycosylase gene polymorphism (Ser326Cys) and cancer risk: updated meta-analysis

**DOI:** 10.18632/oncotarget.16226

**Published:** 2017-03-15

**Authors:** Sang Wook Kang, Su Kang Kim, Hae Jeong Park, Joo-Ho Chung, Ju Yeon Ban

**Affiliations:** ^1^ Kohwang Medical Institute, School of Medicine, Kyung Hee University, Seoul, Republic of Korea; ^2^ Department of Dental Pharmacology, School of Dentistry, Dankook University, Cheonan, Republic of Korea

**Keywords:** *hOGG1*, polymorphism, Ser326Cys, cancer, meta-analysis

## Abstract

Genetic polymorphism of human 8-oxoguanine glycosylase 1 (*hOGG1*) has been reported to have a relationship with the risk of the development of various cancers. Many studies have described the influence of Ser326Cys polymorphism of the *hOGG1* gene on cancer susceptibility. However, the results have remained inconclusive and controversial. Therefore, we performed a meta-analysis to more precisely determine the relationship between the *hOGG1* polymorphism and the development of cancer.

Electronic databases including PubMed, Embase, Google Scholar, and the Korean Studies Information Service System (KISS) were searched. The odds ratio (OR), 95% confidence interval (CI), and p value were calculated to assess the strength of the association with the risk of cancer using Comprehensive Meta-analysis software (Corporation, NJ, USA). The 127 studies including 38,757 cancer patients and 50,177 control subjects were analyzed for the meta-analysis.

Our meta-analysis revealed that G allele of Ser326Cys polymorphism of the *hOGG1* gene statistically increased the susceptibility of cancer (all population, OR = 1.092, 95% CI = 1.051-1.134, *p* < 0.001; in Asian, OR = 1.095, 95% CI = 1.048-1.145, *p* < 0.001; in Caucasian, OR = 1.097, 95% CI = 1.033-1.179, *p* = 0.002). Also, other genotype models showed significant association with cancer (*p* < 0.05, respectively).

The present meta-analysis concluded that the G allele was associated with an increased risk of cancer. It suggested that the *hOGG1* polymorphism may be a candidate marker of cancer.

## INTRODUCTION

Cancers are serious problem around the world and complex, multistep, multifactorial, and highly fatal diseases. The environment and genetic inheritance have been known as risk factor in development of cancer [1]. Several recent studies focused on the genetic background and how the single nucleotide polymorphism (SNP) of specific genes, including DNA damage, can enhance cancer susceptibility [2].

DNA damage plays an important role in tumor development. Reactive oxygen species (ROS) increases damage to DNA and causes miscoding by DNA polymerase [3]. The level of ROS in tissue DNA reflects a balance between the rate of damage and repair. Abnormal balance results in DNA mutations that can activate oncogenes or inactivate tumor suppressor genes, which leads to cancer [4]. The base excision repair (BER) pathway is one of the DNA repair process. An important role of BER is to remove DNA damage caused by various carcinogens, such as ionizing radiation or reactive oxidative species [5]. BER has also evolved to cope with mutagenic and cytotoxic hydrolytic, oxidative, and alkylation damages. A relationship of BER to cancer progression has been drawn from the observation that mutations or altered expression in BER genes [6].

The hOGG1 is a DNA repair enzyme that excises 7,8-dihydro-8-oxoguanine (8oxoG) from DNA. The *hOGG1* is located on chromosome 3p26, a vital member of the BER pathway, and encodes 8-oxoguanine glycosylase that is a key enzyme in the repair of 8-oxoguanine [7]. ROS can lead to mutagenic base 8oxoG formation in DNA and carcinogenesis [8]. Since 8oxoG is a highly mispairing lesion, it was suggested that decreased hOGG1expression level could lead to a higher background mutation frequency and could possibly increase the cancer risk of an individual under oxidative stress [7].

Many previous studies showed the relationship between the Ser326Cys polymorphism of *hOGG1* gene and cancer susceptibility. Meta-analysis on the *hOGG1* polymorphism and the risk of bladder cancer shows no statistically significant association [9]. The meta-analysis on breast cancer suggested that the allele of *hOGG1* 326Cys plays a protective effect in European women but not in different menopausal status (premenopausal and postmenopausal) or the other ethnicities (Asians and Americans) [10]. The *hOGG1* polymorphism may be also contributed to the susceptibility of digestive cancers [11], colorectal cancer [12], esophageal squamous cell carcinoma [13] but shows a lack of association in gastric cancer [14]. In addition, the *hOGG1* polymorphism is associated with hepatocellular carcinoma [15], head and neck cancer [16], and prostate cancer [17], not with lung cancer [18].

Meta-analysis study in 2011 year reported that the evidence of the association between the *hOGG1* polymorphism and cancer risk [19]. Since 2011, many studies reported the relation between the *hOGG1* polymorphism and various cancer risks. However, the results have not been updated yet. Therefore, the purpose of this meta-analysis is to update previous meta-analysis with the aim of elucidating the association of the *hOGG1* polymorphism and risk of cancer.

## RESULTS

**Figure 1:**
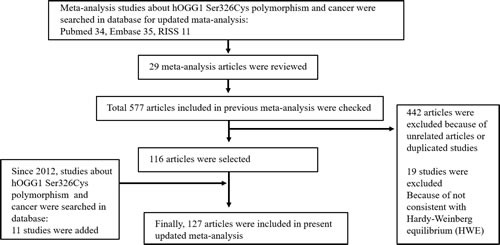
Flow chart illustrating the search strategy used for this meta-analysis to identify studies that examined the association between *hOGG1* Ser326Cys polymorphism and risk of cancer

In present study, we performed the meta-analysis to assess relationship between the *hOGG1* polymorphism and risk of cancer. We collected the genetic data from electronic databases. The search strategy used for this meta-analysis is shown in Figure 1. We examined the 577 articles and 442 articles were excluded as they were unrelated articles or duplicated studies. Among them, 19 studies were excluded because they were not consistent with Hardy-Weinberg equilibrium (HWE). After 116 articles were selected, 11 studies about the *hOGG1* polymorphism since 2012 were added. Finally, a total of 127 genetic studies about the *hOGG1* polymorphism and cancer were analyzed for meta-analysis (Supplementary Table 1) [5, 10–143]. Supplementary Table 1 shows basic characteristics of the analyzed studies. The total 88,934 individuals comprised of 38,757 cancer patients and 50,177 control subjects. The types of cancers were including colorectal (18 articles), lung (28 articles), breast (16 articles), bladder (4 articles), gallbladder (2 articles), prostate (7 articles), gastric (13 articles), esophageal (10 articles), head and neck (8 articles), hepatocellular cancers (7 articles), and etc.

Table 1 presents the results of meta-analysis of association between the *hOGG1* polymorphism and risk of cancer in allele (C *vs*. G), dominant (C/C genotype *vs*. C/G+G/G genotypes), and recessive (C/C+C/G genotypes *vs*. G/G genotype) models. The frequencies of major allele C/minor allele G in the total cancer and control were 63.33%/36.67%and 65.34%/34.66%. The minor G allele frequency in the total cancer was higher more than that of control (36.67% *vs*. 34.66%). The difference showed the significantly strong association with risk of cancer (OR=1.088, 95% CI=1.048-1.130, *p*<0.001 in Table 1). In the subgroup according to type cancer, as shown in Table 1, colorectal cancer, lung cancer, prostate cancer and head and neck cancer presented the association with risk of cancer (colorectal cancer, OR=1.121, 95% CI=1.005-1.251, *p*=0.040; lung cancer, OR=1.094, 95% CI=1.020-1.172, *p*=0.012; prostate cancer OR=1.459, 95% CI=1.068-1.992, *p*=0.018; head and neck cancer, OR=1.335, 95% CI=1.079-1.651, *p*=0.008). The frequencies of CC genotype/CG+GG genotypes in the total cancer and control were 43.17%/56.83% and 45.02%/54.98%. The CG+GG genotypes frequency in the total cancer was higher more than that of control (56.83% *vs*. 54.98%). The difference showed the significant association with risk of cancer (OR=1.075, 95% CI=1.023-1.130, *p*=0.004 in Table 1). In the subgroup according to type cancer, head and neck cancer only showed the association with risk of cancer (head and neck cancer, OR=1.424, 95% CI=1.099-1.845, *p*=0.007). The frequencies of CC+CG genotypes/GG genotype in the total cancer and control were 83.56%/16.44% and 85.69%/14.31%. The CG+GG genotypes frequency in the total cancer was higher more than that of control (16.44% *vs*. 14.31%). The difference showed the significant association with risk of cancer (OR=1.174, 95% CI=1.094-1.259, *p*<0.001 in Table 1). In the subgroup according to type cancer, lung cancer and head and neck cancer presented the association with risk of cancer (lung cancer, OR=1.188, 95% CI=1.055-1.337, *p*=0.004; head and neck cancer, OR=1.551, 95% CI=1.045-2.301, *p*=0.029).

**Table 1:** Overall analysis between *hOGG1* Ser326Cys polymorphism and risk of cancer

Cancers	No. of studies	Heterogeneity		Model	OR (95% CI)	*p*
*p*	I-squared
C *vs*. G						
All cancers	125	<0.001	61. 138	Random	1.092 (1.051-1.134)	**<0.001**
Colorectal cancer	17	<0.001	65.928	Random	1.121 (1.005-1.251)	**0.040**
Lung cancer	28	<0.001	51.835	Random	1.094 (1.020-1.172)	**0.012**
Breast cancer	16	0.077	35.7	Fixed	1.031 (0.985-1.079)	0.185
Bladder cancer	4	0.004	77.187	Random	1.058 (0.812-1.379)	0.676
Gallbladder cancer	2	0.015	83.061	Random	1.044 (0.877-1.242)	0.627
Prostate cancer	6	<0.001	79.676	Random	1.459 (1.068-1.992)	**0.018**
Gastric cancer	13	0.034	46.33	Random	1.011 (0.883-1.157)	0.874
Esophageal cancer	10	0.112	37.044	Fixed	1.050 (0.957-1.152)	0.299
Head and neck cancer	8	<0.001	77.552	Random	1.335 (1.079-1.651)	**0.008**
Hepatocellular cancer	7	<0.001	74.985	Random	1.089 (0.883-1.344)	0.424
Acute lymphoblastic leukemia	2	0.002	89.539	Random	1.579 (0.775-3.217)	0.208
Pancreatic adenocarcinoma	2	0.467	<0.001	Fixed	1.007 (0.885-1.146)	0.917
C/C *vs*. C/G+G/G						
All cancers	127	<0.001	56.428	Random	1.079 (1.027-1.134)	**0.002**
Colorectal cancer	18	<0.001	60.869	Random	1.140 (0.993-1.308)	0.063
Lung cancer	28	0.004	47.036	Random	1.080 (0.984-1.187)	0.106
Breast cancer	16	0.202	22.109	Fixed	1.011 (0.948-1.078)	0.742
Bladder cancer	4	0.39	0.312	Fixed	1.000 (0.847-1.181)	0.996
Gallbladder cancer	2	0.029	78.918	Random	1.080 (0.644-1.812)	0.771
Prostate cancer	7	<0.001	75.606	Random	1.401 (0.976-2.011)	0.067
Gastric cancer	13	0.346	9.913	Fixed	0.928 (0.821-1.048)	0.229
Esophageal cancer	10	0.199	26.588	Fixed	0.971 (0.854-1.104)	0.652
Head and neck cancer	8	<0.001	74.243	Random	1.424 (1.099-1.845)	**0.007**
Hepatocellular cancer	7	<0.001	88.181	Random	1.113 (0.673-1.841)	0.677
Acute lymphoblastic leukemia	2	0.006	86.606	Random	1.401 (0.583-3.364)	0.451
Pancreatic adenocarcinoma	2	0.451	<0.001	Fixed	1.051 (0.898-1.230)	0.539
C/C+C/G *vs*. G/G						
All cancers	125	<0.001	54.586	Random	1.178 (1.098-1.263)	**<0.001**
Colorectal cancer	17	0.04	41.096	Random	1.154 (0.959-1.388)	0.129
Lung cancer	28	0.03	36.264	Random	1.188 (1.055-1.337)	**0.004**
Breast cancer	16	0.378	6.626	Fixed	1.092 (1.004-1.189)	0.041
Bladder cancer	4	<0.001	86.372	Random	1.118 (0.557-2.246)	0.753
Gallbladder cancer	2	0.074	68.567	Fixed	1.099 (0.761-1.585)	0.615
Prostate cancer	6	0.003	72.07	Random	1.691 (0.965-2.965)	0.066
Gastric cancer	13	0.037	46.74	Random	1.088 (0.823-1.438)	0.553
Esophageal cancer	10	0.02	54.195	Random	1.252 (0.929-1.686)	0.139
Head and neck cancer	8	0.009	62.394	Random	1.551 (1.045-2.301)	**0.029**
Hepatocellular cancer	7	<0.001	85.472	Random	1.126 (0.723-1.754)	0.600
Acute lymphoblastic leukemia	2	0.011	84.554	Random	2.435 (0.632-9.376)	0.196
Pancreatic adenocarcinoma	2	0.809	<0.001	Fixed	0.823 (0.578-1.172)	0.280

OR odds ratio, vs versus. Bold numbers indicate significant association with risk of cancer.

**Figure 2:**
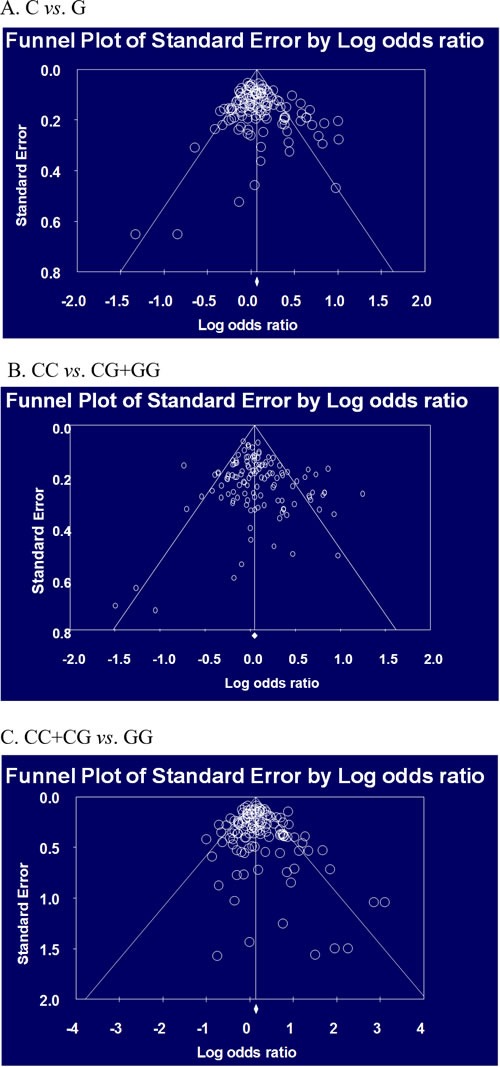
Begg's funnel plot for publication bias in selection of studies on the *hOGG1* Ser326Cys polymorphism (C *vs*. G, C/C *vs*. C/G+G/G, and C/C+C/G *vs*. G/G in all population)

Table 2 and Table 3 present the results of meta-analysis of association between the *hOGG1* polymorphism and risk of cancer according to ethnic difference. In Asian population, analysis of allele, dominant, and recessive models showed the association with risk of cancer (C *vs*. G, OR=1.095, 95% CI=1.048-1.145, *p*<0.001: CC *vs*. CG+GG, OR=1.096, 95% CI=1.015-1.183, p=0.019; CC+CG *vs*. GG, OR=1.171, 95% CI=1.070-1.282, *p*=0.001 in Table 2). According to the type of cancer, risk of lung, breast, and head and neck cancers was associated with the *hOGG1* polymorphism (*p*<0.05, Table 2). In Caucasian population, analysis of allele and recessive models showed the association with risk of cancer (C *vs*. G, OR=1.097, 95% CI=1.021-1.179, *p*=0.012: CC+CG *vs*. GG, OR=1.158, 95% CI=1.005-1.334, *p*=0.043 in Table 3). According to type of cancer, risk of colorectal, esophageal, and head and neck cancer was associated with the *hOGG1* polymorphism (*p*<0.05, Table 3). Begg's funnel plot and Egger's test were used to evaluate publication bias. The results of funnel plots and the Egger's test showed no publication bias in this meta-analysis except for allele model of all cancers (Figure 2). We found a weak publication bias in allele model of all cancers (*p* = 0.03425). In addition, 3 more subgroup analysis showed publication bias (recessive model of head and neck cancer and colorectal cancer in all population; recessive model of all cancer in Caucasian population, data not shown). In sensitivity analysis for our meta-analysis, some results were influenced by some studies. In all population analysis, allele model of lung, prostate, and head and neck cancer, dominant model of all cancers and head and neck cancer, and recessive model of all cancers, lung, and head and neck cancer were not influenced according to sensitivity analysis. In Asian population analysis, allele model of all cancers and lung cancer, dominant model of all cancers and head and neck cancer, and recessive model of all cancers, lung and breast cancer were not influenced. In Caucasian population analysis, allele model of all cancers and dominant model of esophageal cancer were not influenced by studies. These results indicate that individual with minor G allele of the *hOGG1* polymorphism may be increased risk of cancer.

**Table 2:** Overall analysis between *hOGG1* Ser326Cys polymorphism and risk of cancer in Asian

Cancers	Comparison	Heterogeneity		Model	OR (95% CI)	*p*
		*p*	I-squared			
All cancers	C *vs*. G	<0.001	44.278	Random	1.095 (1.048-1.145)	**<0.001**
	CC *vs*. CG+GG	<0.001	48.900	Random	1.096 (1.015-1.183)	**0.019**
	CC+CG *vs*. GG	<0.001	63.487	Random	1.171 (1.070-1.282)	**0.001**
Colorectal cancer	C *vs*. G	0.149	47.481	Fixed	0.987 (0.879-1.108)	0.822
	CC *vs*. CG+GG	0.123	<0.001	Fixed	1.068 (0.868-1.315)	0.532
	CC+CG *vs*. GG	0.451	<0.001	Fixed	0.945 (0.795-1.122)	0.517
Lung cancer	C *vs*. G	0.487	<0.001	Fixed	1.110 (1.048-1.176)	**<0.001**
	CC *vs*. CG+GG	0.163	27.193	Fixed	1.116 (1.013-1.229)	**0.027**
	CC+CG *vs*. GG	0.220	21.505	Fixed	1.176 (1.074-1.289)	**0.000**
Breast cancer	C *vs*. G	0.376	6.340	Fixed	1.085 (1.013-1.162)	**0.019**
	CC *vs*. CG+GG	0.942	<0.001	Fixed	1.098 (0.971-1.241)	0.135
	CC+CG *vs*. GG	0.108	44.685	Fixed	1.122 (1.014-1.242)	**0.026**
Bladder cancer	C *vs*. G	0.018	75.198	Random	1.135 (0.821-1.571)	0.444
	CC *vs*. CG+GG	0.848	<0.001	Fixed	1.157 (0.910-1.472)	0.235
	CC+CG *vs*. GG	<0.001	89.184	Random	1.264 (0.503-3.174)	0.619
Gallbladder cancer	C *vs*. G	0.015	83.061	Random	1.108 (0.710-1.728)	0.652
	CC *vs*. CG+GG	0.029	78.918	Random	1.080 (0.644-1.812)	0.771
	CC+CG *vs*. GG	0.074	68.567	Fixed	1.099 (0.761-1.585)	0.615
Gastric cancer	C *vs*. G	0.075	49.976	Fixed	1.010 (0.904-1.129)	0.855
	CC *vs*. CG+GG	0.561	<0.001	Fixed	0.977 (0.811-1.176)	0.802
	CC+CG *vs*. GG	0.012	65.907	Random	1.232 (0.823-1.843)	0.311
Esophageal cancer	C *vs*. G	0.837	<0.001	Fixed	1.087 (0.980-1.205)	0.113
	CC *vs*. CG+GG	0.733	<0.001	Fixed	1.017 (0.875-1.182)	0.821
	CC+CG *vs*. GG	0.031	56.757	Random	1.277 (0.939-1.735)	0.119
Head and neck cancer	C *vs*. G	0.020	81.661	Random	1.499 (0.729-3.084)	0.271
	CC *vs*. CG+GG	0.274	16.259	Fixed	1.856 (1.262-2.731)	**0.002**
	CC+CG *vs*. GG	0.124	57.636	Fixed	0.964 (0.696-1.335)	0.825
Hepatocellular cancer	C *vs*. G	0.001	74.985	Random	1.089 (0.883-1.344)	0.424
	CC *vs*. CG+GG	<0.001	89.658	Random	1.113 (0.673-1.841)	0.677
	CC+CG *vs*. GG	<0.001	85.472	Random	1.126 (0.723-1.754)	0.600

OR odds ratio, vs versus. Bold numbers indicate significant association with risk of cancer.

**Table 3:** Overall analysis between *hOGG1* Ser326Cys polymorphism and risk of cancer in Caucasian

Cancers	Comparison	Heterogeneity		Model	OR (95% CI)	*p*
		*p*	I-squared			
All cancers	C *vs*. G	<0.001	68.987	Random	1.097 (1.033-1.166)	**0.002**
	CC *vs*. CG+GG	<0.001	62.672	Random	1.078 (1.007-1.154	**0.031**
	CC+CG *vs*. GG	<0.001	45.629	Random	1.183 (1.053-1.331)	**0.004**
Colorectal cancer	C *vs*. G	<0.001	74.844	Random	1.255 (1.053-1.497)	**0.011**
	CC *vs*. CG+GG	<0.001	73.239	Random	1.234 (1.004-1.516)	**0.045**
	CC+CG *vs*. GG	0.036	49.696	Random	1.509 (1.031-2.211)	**0.034**
Lung cancer	C *vs*. G	<0.001	74.319	Random	1.097 (0.920-1.309)	0.303
	CC *vs*. CG+GG	0.001	67.238	Random	1.081 (0.895-1.306)	0.420
	CC+CG *vs*. GG	0.034	50.306	Random	1.175 (0.814-1.696)	0.389
Breast cancer	C *vs*. G	0.117	36.459	Fixed	0.990 (0.932-1.053)	0.756
	CC *vs*. CG+GG	0.075	42.399	Fixed	0.980 (0.909-1.056)	0.596
	CC+CG *vs*. GG	0.728	<0.001	Fixed	1.026 (0.880-1.197)	0.739
Prostate cancer	C *vs*. G	<0.001	90.407	Random	1.397 (0.702-2.780)	0.342
	CC *vs*. CG+GG	0.001	85.774	Random	1.503 (0.734-3.078)	0.265
	CC+CG *vs*. GG	<0.001	87.260	Random	1.604 (0.344-7.479)	0.548
Gastric cancer	C *vs*. G	0.081	55.491	Fixed	0.889 (0.752-1.050)	0.166
	CC *vs*. CG+GG	0.131	56.157	Fixed	0.880 (0.722-1.073)	0.207
	CC+CG *vs*. GG	0.467	<0.001	Fixed	0.802 (0.498-1.292)	0.364
Esophageal cancer	C *vs*. G	0.149	52.096	Fixed	0.660 (0.482-0.904)	0.148
	CC *vs*. CG+GG	0.102	51.614	Fixed	0.627 (0.434-0.905)	**0.013**
	CC+CG *vs*. GG	0.943	<0.001	Fixed	0.427 (0.144-1.263)	0.124
Head and neck cancer	C *vs*. G	<0.001	83.256	Random	1.396 (1.001-1.946)	**0.049**
	CC *vs*. CG+GG	0.001	79.552	Random	1.406 (0.975-2.027)	0.068
	CC+CG *vs*. GG	0.014	67.859	Random	2.037 (1.047-3.960)	**0.036**
Pancreatic adenocarcinoma	C *vs*. G	0.562	<0.001	Fixed	1.007 (0.885-1.146)	0.917
	CC *vs*. CG+GG	0.451	<0.001	Fixed	1.051 (0.898-1.230)	0.539
	CC+CG *vs*. GG	0.809	<0.001	Fixed	0.823 (0.578-1.172)	0.280

OR odds ratio, vs versus. Bold numbers indicate significant association with risk of cancer.

## DISCUSSION

It was suggested that cancer susceptibility could result from the interaction of genetic background. Exposure and reductions in DNA repair capacity by common genetic variation affect cancer predisposition [6]. The hOGG1 is generally involved in DNA repair, and has been studied extensively on its relationship with various types of cancer. Low OGG activity in peripheral blood mononuclear cells increases risk of lung cancer [144]. Also, lower expression of hOGG1 mRNA and hOGG1 protein decreases mitochondrial DNA repair to oxidative damage in lung cancer cells [145]. Immunohistochemical expressions in diffuse-type adenocarcinoma of gastric cardia showed lower expression of OGG1, which related to higher T-stage, lymphatic invasion, and lymph node metastasis [146]. A previous study on lung cancer patients showed a close relationship between Ser326Cys polymorphism and OGG1 mRNA levels [57]. The Ser326Cys polymorphisms have been shown to be associated with delayed repair of oxidative DNA damage [147]. In a recent study, changes in the functional and structural characteristics of the hOGG1 protein by the Ser326Cys polymorphism using in silico computational biology tools have been reported. According to this study, *hOGG1* 326Cys variant is smaller and more hydrophobic than wild type, which can have deleterious effects on the function of the hOGG1 protein. And this variant has been found to be closely related to breast cancer [143]. Although the relationship between hOGG1 expression and cancer risk and the Ser326Cys *hOGG1* polymorphism and the expression of OGG1 has been reported, the results of previous genetic studies on the relationship between *hOGG1* polymorphism and various cancers risks were conflicting and contradictory. This meta-analysis was performed to provide a quantitative approach to for the different results.

Meta-analysis on the *hOGG1* polymorphism and the risk of bladder cancer, gastric cancer, and lung cancer shows no statistically significant association. But the other meta-analysis reported that the *hOGG1* polymorphism may contribute to the susceptibility of digestive cancers, breast cancer, colorectal cancer, esophageal squamous cell carcinoma, hepatocellular carcinoma, head and neck cancer, and prostate cancer. Among the previous meta-analysis studies, inappropriate data was included in the analysis. Therefore, there was error. For example, Zhu et al. 2012 investigated whether the *hOGG1* polymorphism was associated with prostate cancer using meta-analysis [17]. The meta-analysis included genotype data of rs3218997 SNP of *OGG1* that reported by Agalliu et al. 2010 [148]. Because rs3218997 SNP is different from Ser326Cys of *hOGG1*, the genetic data had to be excluded for the exact meta-analysis. In addition, some previous studies included articles which were not consistent with HWE. We evaluated HWE in all the articles, some articles were excluded. So, we performed this meta-analysis to combine and update from the different results.

In present study, total of 126 genetic studies about the *hOGG1* polymorphism and cancer were analyzed for meta-analysis. Significant relationship between the *hOGG1* polymorphism and overall cancer risk was found. In subgroup analyses by cancer types, the significant association between the *hOGG1* polymorphism and colorectal, lung, prostate, and head and neck cancer risk was detected. In addition, in subgroup analyses by ethnicities, we found that the *hOGG1* polymorphism was significantly associated with overall cancer risk in both Caucasian and Asian population. But there was a little different result between Asian and Caucasian population. In Asian population, lung, breast, and head and neck cancer showed a relation with the *hOGG1* polymorphism but in Caucasian population only head and neck cancer showed. On the other hands, an association with colorectal cancer and esophageal cancer was only shown in Caucasian population. Some our results were consistent with or contrary to previous meta-analysis. Overall cancer risk and the *hOGG1* polymorphism was significantly associated in our results (allele, *p*<0.001; dominant, *p*=0.004; recessive, *p*<0.001) and previous study showed similar result [19]. Results of previous meta-analysis on lung cancer were different from our present results [18]. Our results showed the statistically significance in lung cancer (allele and recessive model in overall analysis, allele, dominant, recessive model in Asian). It is seemed that it is because the previous study included some articles which were not in HWE in their meta-analysis. Meta-analysis on colorectal cancer was consistent with ours [12]. The *hOGG1* polymorphism had a connection with the colorectal cancer risk among the total population, and especially among Caucasians. One meta-analysis on breast cancer reported the association between the *hOGG1* polymorphism and breast cancer risk [10] but another study suggested a lack of association [149]. Our meta-analysis showed the different results. An association between the *hOGG1* polymorphism and breast cancer risk was found only in Asian population (allele, *p*=0.019; recessive model, *p*=0.026). Not only previous meta-analysis on bladder, gallbladder, and gastric cancer risk but also our present meta-analysis showed no statistically significance [9, 11, 14]. Zhang et al. reported the relation between the *hOGG1* polymorphism and esophageal cancer risk [13] but meta-analysis by Wang showed no association [11]. In our study, an association between the *hOGG1* polymorphism and esophageal cancer risk was found in dominant model in Caucasian population (*p*=0.013). Previous meta-analysis reported that the *hOGG1* polymorphism had a relation to hepatocellular cancer [15]. However, we could not find an association in any model. The results of previous meta-analysis on prostate cancer consisted with ours [17] but we could not find any association in Caucasian population and some studies influenced the results according to sensitivity analysis. Similar to previous study, our study showed the relation between the *hOGG1* polymorphism and head and neck cancer [16]. But recessive model in all population analysis showed publication bias and several results except for allele and dominant model in all population analysis and dominant model in Asian population analysis were influenced by some studies according to sensitivity analysis.

From dbSNP database, The C and G allele frequencies have been reported to be 0.776 and 0.224 in European, 0.500 and 0.500 in Chinese, 0.477 and 0.523 in Japanese, and 0.856 and 0.144 in Sub-Saharan African populations, respectively. And the CC, CG and GG genotype frequencies have been reported to be 0.621, 0.310, and 0.069 in European, 0.244, 0.511, and 0.244 in Chinese, 0.182, 0.591, and 0.227 in Japanese, and 0.746, 0.220, and 0.034 in Sub-Saharan African populations, respectively. In our results, the C and G allele frequencies have been reported to be 0.762 and 0.238 in Caucasian and 0.484 and 0.516 in Asian. And the CC, CG and GG genotype frequencies have been shown to be 0.584, 0.356, and 0.060 in Caucasian and 0.246, 0.477, 0.277 in Asian. We found that the genotype and allele frequencies in Caucasian and Asian showed significant difference, which might affect the roles of the *hOGG1* polymorphism on cancer risk in Asians and Caucasians.

This meta-analysis has several limitations. Our results showed the genetic difference and different cancer risks in ethnicity but included studies regarded only Caucasians and Asians, but not other races like African. Because of limited data, we simply divided the ethnicity into Asian and Caucasian. The genetic heterogeneity plays an important role in the carcinogenesis but it is an interaction between environment factors and genetic background. This analysis could not reflect environmental exposures. And there were considerable inadequate data in previous studies, especially in meta-analysis. We had examined the included articles as many as possible such as HWE or data in the article but there could be still omitted data. And several results showed significant associations but revealed publication bias.

## CONCLUSIONS

In spite of some limitations, this meta-analysis could provide the evidence of the strong association between the *hOGG1* polymorphism and cancer risk. In summary, G allele of Ser326Cys polymorphism might play a role in the carcinogenesis and the genotype and allele frequencies difference makes the ethnicity difference in carcinogenesis. If further study with large sample size in diverse ethnic populations were performed, it would provide more precise understanding of the association between the *hOGG1* polymorphism and various cancer risks. This SNP could be a candidate of biomarker for cancer screening, diagnosis, and therapy in the future.

## MATERIAL AND METHODS

### Search strategy

In order to select eligible studies about the *hOGG1* polymorphism and cancer, electronic database including Pubmed, Embase, google of scholar, and KISS were investigated up to April 2015. We searched meta-analysis study about the *hOGG1* polymorphism and also searched the association study between the *hOGG1* polymorphism and risk of cancer. The keywords to find these studies were following: “8-oxoguanine DNA glycosylase”, “hOGG1“, or “DNA repair gene”, AND “polymorphism”, “polymorphisms”, or “variant” AND “Ser326Cys” AND “cancer or carcinoma”, or “meta analysis”. The previous meta-analysis studies about the *hOGG1* polymorphism and cancer were considered as reference.

### Inclusion criteria and data extraction

Selected studies were included in the meta-analysis if they met the following criteria: (1) Investigated the association study between the *hOGG1* polymorphism and cancer; (2) A comparison between cancer and control; (3) Included genotype and allele distributions of Ser326Cys polymorphism for genetic analysis. The data of first author's name, year of publication, country of origin, ethnicity of study population, sample size of cancer and control, and genotype frequencies of the *hOGG1* polymorphism in cancer and control were extracted from the final selected studies. The allele distributions were calculated from genotype distributions in the cancer group and the control group. The ethnicity was divided into Asian and Caucasian.

### Statistical analysis

HWE in all include studies was tested by the Chi-square test. Meta-analysis was performed using the Comprehensive Meta-analysis software. The pooled p value, OR, and 95% CI were used to assess the strength of association between risk of cancer and the *hOGG1* polymorphism. All the results were re-analyzed to see the effect of each paper on the final results by sensitivity analysis. The meta-analysis was repeated while omitting each study one at a time to examine the influence of each study on the pooled OR. For the regression analysis in this meta-analysis, the random effects model or the fixed effects model was used. OR with the corresponding 95 % CI was calculated for the dominant model (C/C + C/G genotypes *vs*. G/G genotype) and recessive model (C/C *vs*. C/G + G/G genotypes), and allele (C *vs*. T), respectively [150,151]. The *p*<0.05 was regarded as statistically significant. A χ2-test-based Q statistic test was used to assess heterogeneity among studies. We also performed the effect of heterogeneity by *I*2 test. The random-effects Mantel–Haenszel method was adopted if the result of the Q test was p<0.05 or *I*2 statistic was >50 %, which indicated the statistically significant heterogeneity between the studies. Otherwise, the fixed-effects Mantel–Haenszel method was adopted. When more than 3 studies were included, Begg's funnel plot and Egger's test were performed to evaluated publication bias.

## SUPPLEMENTARY MATERIALS TABLE


